# Model-driven discovery of long-chain fatty acid metabolic reprogramming in heterogeneous prostate cancer cells

**DOI:** 10.1371/journal.pcbi.1005914

**Published:** 2018-01-02

**Authors:** Igor Marín de Mas, Esther Aguilar, Erika Zodda, Cristina Balcells, Silvia Marin, Guido Dallmann, Timothy M. Thomson, Balázs Papp, Marta Cascante

**Affiliations:** 1 Department of Biochemistry and Molecular Biomedicine, Faculty of Biology, Universitat de Barcelona, Barcelona, Spain; 2 Institute of Biomedicine of University of Barcelona (IBUB) and Associated Unit with CSIC, Barcelona, Spain; 3 Synthetic and Systems Biology Unit, Institute of Biochemistry, Biological Research Center of the Hungarian Academy of Sciences, Szeged, Hungary; 4 Department of Cell Biology, Barcelona Institute for Molecular Biology (IBMB), National Research Council (CSIC), Barcelona, Spain; 5 Biocrates Life Sciences AG, Innsbruck, Austria; University of California San Diego, UNITED STATES

## Abstract

Epithelial-mesenchymal-transition promotes intra-tumoral heterogeneity, by enhancing tumor cell invasiveness and promoting drug resistance. We integrated transcriptomic data for two clonal subpopulations from a prostate cancer cell line (PC-3) into a genome-scale metabolic network model to explore their metabolic differences and potential vulnerabilities. In this dual cell model, PC-3/S cells express Epithelial-mesenchymal-transition markers and display high invasiveness and low metastatic potential, while PC-3/M cells present the opposite phenotype and higher proliferative rate. Model-driven analysis and experimental validations unveiled a marked metabolic reprogramming in long-chain fatty acids metabolism. While PC-3/M cells showed an enhanced entry of long-chain fatty acids into the mitochondria, PC-3/S cells used long-chain fatty acids as precursors of eicosanoid metabolism. We suggest that this metabolic reprogramming endows PC-3/M cells with augmented energy metabolism for fast proliferation and PC-3/S cells with increased eicosanoid production impacting angiogenesis, cell adhesion and invasion. PC-3/S metabolism also promotes the accumulation of docosahexaenoic acid, a long-chain fatty acid with antiproliferative effects. The potential therapeutic significance of our model was supported by a differential sensitivity of PC-3/M cells to etomoxir, an inhibitor of long-chain fatty acid transport to the mitochondria.

## Author summary

The coexistence within the same tumor of a variety of subpopulations, featuring different phenotypes (intra-tumoral heterogeneity) represents a challenge for diagnosis, prognosis and targeted therapies. In this work, we have explored the metabolic differences underlying tumor heterogeneity by building cell-type-specific genome-scale metabolic models that integrate transcriptome and metabolome data of two clonal subpopulations derived from the same prostate cancer cell line (PC-3). These subpopulations display either highly proliferative, cancer stem cell (PC-3/M) or highly invasive, epithelial-mesenchymal-transition-like phenotypes (PC-3/S). Our model-driven analysis and experimental validations have unveiled a differential utilization of the long-chain fatty acids pool in both subpopulations. More specifically, our findings show an enhanced entry of long-chain fatty acids into the mitochondria in PC-3/M cells, while in PC-3/S cells, long-chain fatty acids are used as precursors of eicosanoid metabolism. The different utilization of long-chain fatty acids between subpopulations endows PC-3/M cells with a highly proliferative phenotype while enhances PC-3/S invasive phenotype. The present work provides a tool to unveil key metabolic nodes associated with tumor heterogeneity and highlights potential subpopulation-specific targets with important therapeutic implications.

## Introduction

Prostate cancer (PC) is the most commonly diagnosed non-cutaneous malignancy among Western men and accounts for the second leading cause of cancer-related death [1]. In the majority of cases, PC eventually becomes independent of androgens, resuming growth after androgen-deprivation therapies in a more aggressive and therapy-refractory form [2].

The coexistence within the same tumor of a variety of cell subpopulations, featuring different phenotypes (intra-tumoral heterogeneity) associated with tumor evolution and progression reflects extreme plasticity and adaptation capability of neoplastic cells. This diversity is reached through genetic evolution of neoplastic cells and epigenetic and metabolic reprogramming of neoplastic and non-neoplastic tumor components that enhance tumor progression and represent a challenge for targeted therapies [3,4].

A major driver of intra-tumor heterogeneity is Epithelial-Mesenchymal transition (EMT), which induces alterations in the intricate and large cancer cell gene regulatory and metabolic networks (metabolic reprogramming) [5]. However, although EMT-mediated molecular and cellular changes have been widely studied, the EMT-induced metabolic changes remain poorly understood. In this sense, it is widely accepted that metabolic reprogramming is one of the ten hallmarks of cancer [6] which endows cancer cells with a phenotype characterized by a rapid and continuous proliferation, metastasis, invasion, and treatment resistance. Thus, study of the metabolism in these heterogeneous cellular populations is of special interest and must be approached from a global perspective integrating global metabolism with consideration of different subpopulations.

In this context, integration of omics data from high-throughput technologies, such as transcriptomics, into a genome-scale metabolic network reconstruction analysis, has been successfully used to study the metabolic mechanisms underlying different cancer types [7,8]. However, the differences in metabolic physiology between intra-tumoral subpopulations have not yet been taken into account in these computational approaches.

Here, we have built comparative genome-scale metabolic network models based on transcriptomic data for two clonal sub-populations isolated and separated from an established prostate cancer cell line (PC-3): i) a Cancer Stem Cell subpopulation -CSC- with high metastatic potential, low invasiveness and a higher proliferation rate (PC-3/M cells) and ii) a non-CSC subpopulation expressing EMT markers with high invasiveness and low metastatic potential (PC-3/S cells) [9]. These neoplastic cell sub-populations, capturing extreme epithelial *vs*. mesenchymal phenotypes, were derived from the same tumor cell line and represent an excellent cellular model to study how intra-tumoral heterogeneity and the different phenotypes endowed by the different subpopulations provides advantages to the tumor in terms of metastatic capability and drug resistance.

Our computational analysis has unveiled several subpopulation-specific metabolic alterations associated with long-chain fatty acids (LCFA) metabolism. First, we have identified an increased transport activity of LCFA into the mitochondria via Carnitine palmitoyl transferase I (CPT1), suggesting an increased β-oxidation, which could enhance proliferation in the PC-3/M subpopulation. Second, PC-3/S cells are predicted to have enhanced conversion of LCFA to arachidonic acid (AA), the precursor of a variety of eicosanoids that enhance angiogenesis, cell adhesion and invasion. Finally, the lower CPT1 activity predicted in PC-3/S cells leads to Docosahexaenoic acid (DHA) accumulation, a LCFA with antiproliferative effects [10]. The latter prediction is consistent with the lower proliferation rate observed in PC-3/S cells.

Next, using targeted metabolomics measurements, we experimentally confirmed these predictions and demonstrated that: i) the low proliferative rate of PC-3/S cells compared with PC-3/M subpopulation is consistent with higher intracellular concentrations of DHA ii) PC-3/M cells presents a higher CPT1 and β-oxidation activity that can be associated with their observed higher proliferative rate and iii) in PC-3/S, the reported cell adhesion and enhanced invasive capability can be explained by higher levels of AA and eicosanoids, PGE2 and 12S-HETE. Finally, we experimentally showed that the low efficacy of etomoxir (a CPT1 inhibitor) in metastatic PC tumors is conferred by the low sensitivity of non-metastatic subpopulation (PC-3/S) towards this drug in contrast with the high sensitivity showed by metastatic subpopulation (PC-3/M) and can be explained by altered LCFA transport activity facilitated by CPT1. The approach presented hereby provides a tool to unveil key metabolic nodes and vulnerabilities specific to distinct cancer cell subpopulations and opens new avenues in the development of more specific and efficient anti-tumoral therapies.

## Results

### Metabolic network analysis unveils marked differences in LCFA metabolism between PC-3 subpopulations

To infer the activity states of the metabolic networks of PC-3/S and PC-3/M subpopulations, we used previously generated transcriptomic data for PC-3/S and PC-3/M cells based on microarray technology [9] which was integrated into a genome-scale reconstruction of the human metabolic network [11]. In brief, this integrative method defines an upper threshold above which the genes are considered highly expressed and a lower threshold below which the genes are considered lowly expressed and seeks a network activity state in which the number of active reactions associated with highly expressed genes and the number of inactive reactions associated with lowly expressed genes are maximized [12,13]. In other words, this approach defines an objective function intended to maximize the similarity between gene expression and the activity state of the metabolic network rather than predefine an objective function that may not properly describe the cellular phenotype (i.e. biomass maximization). Next, we identified a set of reactions whose activity state was unambiguously different between subpopulations using sensitivity analysis (see Methods and Supplementary information S1 File and S1 Text).

**Fig 1:**
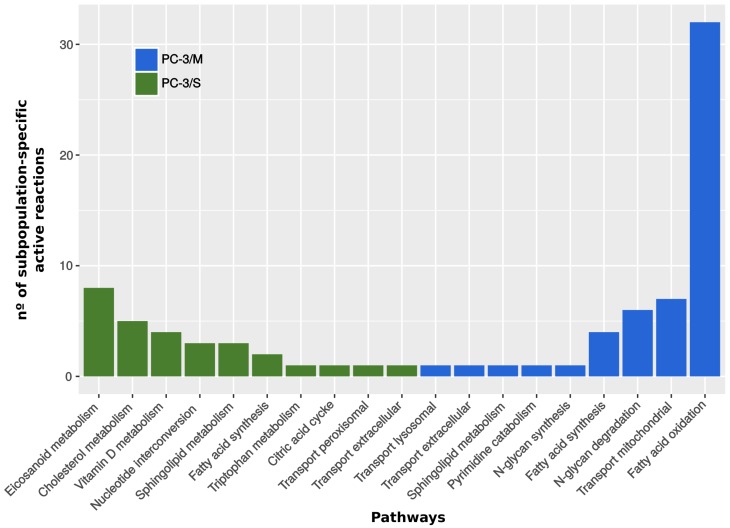
Subpopulation-specific active metabolic reactions and their associated metabolic pathways: This figure illustrates the number of reactions active only in one of the subpopulations and the corresponding pathway. Blue bars are the active reactions in PC-3/M cells and inactive in PC-3/S cells. Green bars are the reactions active in PC-3/S cells and inactive in PC-3/M cells. The p-value associated to the significance of reaction activity prediction is below 0.01 (Supplementary information S2 File).

Finally, our computational analysis permitted to infer, for each subpopulation, a set of active reactions, either intracellular or nutrients uptake/secretion between cell and media (see Fig 1 and Supplementary information S1 Text). The predicted metabolic uptake/secretion rates were in accordance with experimental measurements (true positive rate of 70% with an associated p-value < 0.001; [14]). For instance, our model-driven analysis successfully predicted the consumption / production patterns of most of the bioactive amino-acids, as well as glucose consumption and lactate production in both subpopulations (Supplementary information S1 Text).

This model-driven analysis revealed two major differences between the two subpopulations. First, the activity of fatty acid oxidation predicted by the model is higher in PC-3/M than in PC-3/S cells. The oxidation of fatty acids in the mitochondria produces NADH, FADH_2_ and acetyl-CoA that fuels the production of energy via TCA cycle and the electron transport chain. Most of the reactions differentially activated in this pathway involve carnitine palmitoyl transferase 1 (CPT1). This mitochondrial membrane protein actively transports LCFA from cytosol into the mitochondria [15]. Our analysis provided a set of eight cytosolic LCFAs that were predicted to be substrates of CPT1 exclusively in PC-3/M cells. Interestingly, it has been reported that five of these eight LCFAs, including DHA, have antiproliferative effects [15–20]. Second, the analysis predicted an increased activity of eicosanoid metabolism in PC-3/S cells that is associated with angiogenesis, cell invasion and adhesion [21–23]. Arachidoic acid metabolism (AA) is the main precursor of this pathway that in turn is fueled by a fraction of the LCFA previously mentioned. Finally, our analysis revealed other significant differences between the metabolic activities of the two PC-3 subpopulations that are consistent with published evidences. For example, Vitamin D3 metabolism was predicted to be more active in the PC-3/S subpopulation. This molecule controls proliferation in prostate cells [24] and has antiproliferative effects on a number of cancer cell lines, being PC-3 cells (parental cell line) one of the few cell lines insensitive to this drug [25]. Taken together, the *in silico* analysis suggests the occurrence of metabolic alterations that correlate with a more proliferative phenotype in PC-3/M cells and with a less proliferative and more invasive phenotype in PC-3/S cells. These predictions are consistent with the reported phenotypes of both PC3 subpopulations [9].

### Differential fatty acid uptake into mitochondria may explain the higher proliferative rate and etomoxir sensitivity of PC-3/M cells

**Fig 2:**
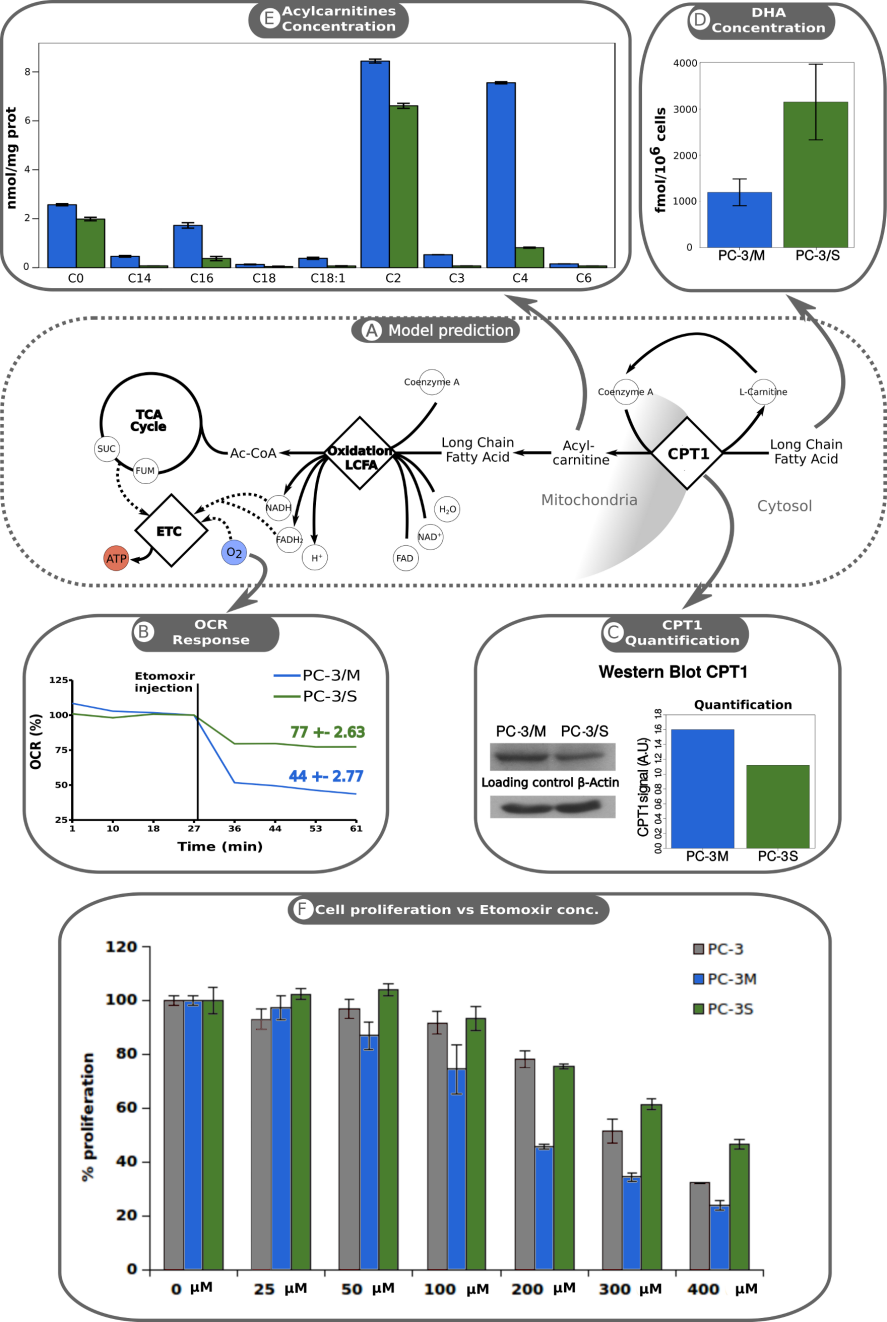
Differences in LCFA metabolism activity between the PC-3/M and PC-3/S cells. A: Computational analysis predicts long chain fatty acid transport (LCFA) from cytosol into the mitochondria via CPT1 and LCFA β-oxidation to be more active in PC-3/M cells. B: PC-3/M cells show a higher sensitivity to CPT1 inhibition. Validation of model predictions by measuring Oxygen consumption rate (OCR) before and after inhibition of CPT1 with etomoxir. Measurement values were normalized to pre-inhibition OCR values. Green line, OCR associated with PC-3/S cells; blue line, OCR associated with PC-3/M cells (mean value of three replicates). The end-point values are represented as means ± SD. p-value < 0.001, calculated using Mann-Whitney U test. C: PC-3/M cells present higher levels of CPT1 protein. Validation of model prediction by measuring CPT1 protein levels by western blotting. β-Actin levels were used as a protein loading and transfer control. On the right, quantification of western blot by using ImageJ software [33]. D: The concentration of DHA, a LCFA with anti-proliferative properties, is significantly higher in PC-3/S cells (p-value < 0.05 calculated with one tail Mann-Whitney U test). Values represent means ± sd of three replicates. PC-3/S: 3143.38 ± 857.98 fmol/10^6 cells; PC-3/M: 1195.91 ± 219.19 fmol/10^6 cells. E: PC-3/M cells present significantly higher levels of acylcarnitines (p-value < 0.001). F: Dose-effect relationship between the antiproliferative effects of etomoxir and cell proliferation in PC-3/M and PC-3/S subpopulations and parental PC-3 cells.

Our computational analysis predicts both a higher LCFA entry into the mitochondria via CPT1 and a more active LCFA β-oxidation in PC-3/M cells. LCFA must be imported into mitochondria to be degraded via β-oxidation and CPT1, a mitochondrial membrane enzyme that plays a critical role in its transport into the mitochondrial matrix [15,26]. To experimentally verify that CPT1 protein levels differ between PC-3/M and PC-3/S cells, we compared CPT1 levels in PC-3/M and PC-3/S subpopulations by western blotting (Fig 2C). In line with the computational inference, we found that PC-3M cells express 30% higher levels of CPT1 than PC-3/S cells. As CPT1 mRNA levels are not significantly different between the two subpopulations (7.68±0.22 A.U in PC-3/M cells and 7.13±0.033 A.U. in PC-3/S cells and p-value > 0.01 using T-test), this highlights the power of the data integration approach to infer metabolic alterations even if mRNA and protein-level changes do no match owing to post-transcriptional regulation [13].

To experimentally determine whether β-oxidation activity is higher in PC-3/M, we measured acylcarnitine levels. These molecules are CPT1 activity intermediates in the entry of acyl‐CoAs into the mitochondrial matrix and are used to experimentally infer the β-oxidation activity [27]. Here we found that acylcarnitine levels were significantly higher in PC-3/M compared with PC-3/S cells (Fig 2E and Supplementary information S6 File), which is in accordance with our computational predictions and supports the hypothesis that PC-3/M cells present a more active β-oxidation. Etomoxir is a CPT1 inhibitor that consequently inhibits β-oxidation and its associated oxygen consumption [15, 26]. Since in many cancer types, tumor onset and progression relies more on lipid fuel than on aerobic glycolysis, this compound is widely used in cancer research [15] but not in clinical practice due to its hepatotoxicity. However, as stated above, its antiproliferative efficacy on PC-3 cells is the lowest among the different PC cell lines studied [15]. Based on these evidences and the results of our analysis, we hypothesized that this could be explained by a low activity of CPT1 in the PC-3/S subpopulation.

To experimentally test this hypothesis, we measured the oxygen consumption rate (OCR) before and after exposure to etomoxir of both subpopulations (Fig 2B, see Methods). We found that PC3/M cells show a 30% higher sensitivity to CPT1 inhibition than PC-3/S cells, implying that CPT1 and hence β-oxidation is more rate-limiting in the PC-3/M subpopulation.

In addition, we studied the dose-effect relation between etomoxir and the proliferation of PC-3/M, PC-3/S and parental PC-3 cells. This experiment also showed a higher sensitivity of PC-3/M cells towards the antiproliferative effects of etomoxir and the difference in the proliferative rate between subpopulations increased with higher concentrations of etomoxir (Fig 2F and Supplementary information S5 File). These experimental observations are in accordance with our computational predictions. Furthermore, PC-3 cells that represent a heterogeneous population containing cells with “PC-3/M-like” and “PC-3/S-like” phenotypes showed an intermediate sensitivity which supports our hypothesis that the low sensitivity of PC-3 cells to etomoxir may be conferred by tumor cell subpopulations with PC-3/S-like metabolic features.

We also hypothesized that, as a side effect of the relatively low CPT1 activity in PC-3/S cells, the levels of some LCFAs would be higher in this subpopulation compared to PC-3/M cells. More specifically, we focused on determining the levels of docosahexaenoic acid (DHA), a compound with antiproliferative effects in cancer [28–31]. To this aim, we used a targeted approach based on the Biocrates platform Assay [32] to quantitatively measure the concentration of DHA in both PC-3 subpopulations. We found that the concentration of DHA was 262% higher in PC-3/S cells compared to PC-3/M cells (Fig 2D, see Methods), which supports our model-driven predictions and is consistent with the lower proliferation rate reported in the PC-3/S subpopulation.

### Differential arachidonic acid and eicosanoid metabolism may explain the lower proliferative rate and high invasiveness of PC-3/S

**Fig 3:**
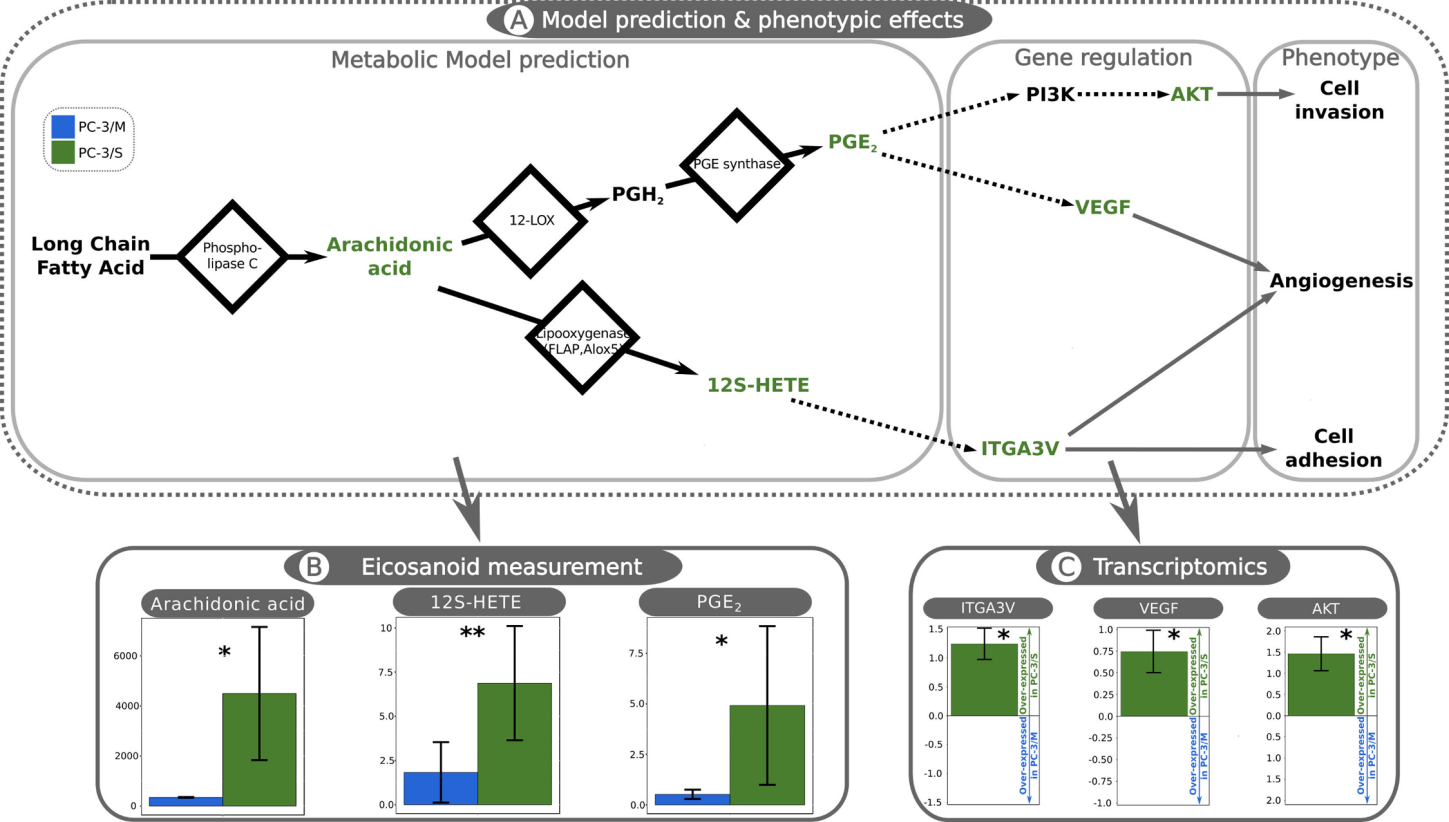
Metabolomic measurements reveal major differences in eicosanoid metabolism between PC-3/M and PC-3/S cells. A: Computational predictions of Eicosanoid metabolism and reported effects on tumor progression involving several gene regulatory mechanisms. The computational analysis predicts a more active eicosanoid metabolism in PC-3/S cells. The left-most box (Metabolic Model prediction) represents a set of eicosanoid metabolism intermediates with significant differences between subpopulations and their associated reactions. Black solid arrows, metabolic reactions; nodes, metabolites; green highlight, measured metabolites. Long-chain fatty acids, Arachidonic acid, 12S-HETE: 12(S)-hydroxy-5Z,8Z,10E,14Z-eicosatetraenoic acid, PGH2: Prostaglandin H2, PGE2: Prostaglandin E2. The central box (Gene regulation) illustrates the gene regulatory interactions associated with eicosanoid metabolism. Green highlight, measured genes; black non-continuous arrows, gene regulatory pathways. *ITGA3V*: Integrin alpha v3; *VEGF*: Vascular Endothelial Growth Factor; *PI3K*: Phosphoinositide 3-kinase; *AKT*. Right panel (phenotype): reported effects on tumor progression, connected to the associated metabolite or gene through gray solid arrows. B: Metabolic measurements of detected species in both PC-3/M (blue bars) and PC-3/S cells (green bars). Shown are mean values ± sd. The units are in fmol/106cells. C: Transcript levels of genes associated with eicosanoid metabolism and tumor progression. The figure represents the mean value of log2 FC between PC-3/S (green bars) and PC-3/M (blue bars) ± sd. Both, metabolite level and gene expression were determined by measuring three independent samples. The level of significance was calculated using the Wilcoxon-Mann-Whitney U test, where p-values < 0.05 are indicated as “*” and < 0.1 as “**”.

A further key prediction of our model-driven analysis is that the eicosanoid metabolism is more active in PC-3/S cells. Arachidonic acid (AA) is the precursor of this pathway, which in turn is metabolized from some of the eight LCFAs that are predicted to be transported by CPT1 into the mitochondria exclusively in PC-3/M cells. Based on these results, we hypothesized that the levels of AA and other eicosanoids were higher in PC-3/S than in PC-3/M cells. To validate this hypothesis, we applied a targeted approach using the Biocrates platform Assay [32], which allows quantitative measurements of arachidonic acid, eicosanoids and other oxidation products of polyunsaturated Fatty Acids (PUFAs). Among all the metabolites measured by this platform (Supplementary information S4 File), AA, Prostaglandin E2 (PGE2) and 12(S)-hydroxy-5Z,8Z,10E,14Z-eicosatetraenoic acid (12S-HETE) were significantly higher in PC-3/S than in PC3-M cells (Fig 3). 12-HETE is the dominant AA metabolite in PC3 cells and its levels in human prostate cancer tissues exceed by > 9-fold its levels in normal human prostate tissue [34]. Furthermore, in PC3 cells, 12(S)-HETE increases the expression of *ITGA3V* gene, which is associated with cell adhesion [20] and promotes PGE2 metabolism in cultured PC3 cells [35]. High PGE2 levels are associated with cancer [36–38] and affects different mechanisms that have been shown to play a role in carcinogenesis such as cell invasion via the Akt signaling pathway [22] or angiogenesis by over-expressing the *VEGF* gene [23,39]. Consistent with these regulatory effects of AA metabolites, the expression level of *ITGA3V*, *VEGF* and *AKT* genes were significantly higher in PC-3/S than in PC-3/M cells (Log_2_ FC of 1.24 ± 0.27, 0.79 ± 0.25, 1.46 ± 0.4 respectively). Taken together, these results indicate a higher activity of eicosanoid metabolism in PC-3/S cells leading to higher levels of eicosanoid pathway intermediates and the upregulation of genes associated with angiogenesis, cell adhesion and invasion, all of which are consistent with the phenotype observed in this subpopulation.

## Discussion

Intratumoral heterogeneity is key to understanding the hierarchical and functional relationships between different neoplastic cell populations within a given tumor, with direct implications on tumor dynamics and progression [40]. Here we have focused on the study of the metabolic profiles of two clonal cell sub-populations isolated from an established prostate cancer cell line (PC-3): PC-3/M and PC-3/S cells [9]. These sub-populations were derived from the same PC cell line and thus they can be assumed to coexist within the same tumor, representing an excellent model to study how intra-tumoral heterogeneity benefits the tumor in terms of invasiveness, metastatic capability and drug resistance. This fact also enables us to investigate the relationships between gene expression and metabolism with tumor-initiating cell or mesenchymal-like attributes in neoplastic cells.

Here, we inferred the metabolic activity states of these PC-3 subpopulations by integrating transcriptomic data with a genome-scale metabolic network reconstruction. The applied constraint-based method treats gene expression levels merely as cues for the likelihood that their associated reactions carry metabolic fluxes and hence allowing for potential post-transcriptional regulatory effects. For example, a reaction associated with a highly expressed gene does not necessarily entail a high flux. As a consequence, the method allows us to infer metabolic activity patterns that go beyond conventional gene expression analysis. Indeed, some of the inferred metabolic differences between PC-3/M and PC-3/S cells do not correlate with the expression patterns of the underlying genes. One example is provided by the expression levels of genes associated with the fatty-acyl-ACP hydrolase reaction that participates in the oxidation of fatty acids. The transcriptomic profiles suggest that these genes are more active in PC-3/S cells, which is in contrast to our computational analysis identifying the corresponding reaction to be active only in the PC-3/M subpopulation (See Supplementary information S3 File). Importantly, we have provided experimental support for this prediction by observing a significantly higher sensitivity of PC-3/M cells to CPT1 and β-oxidation inhibition by etomoxir compared to PC-3/S cells, thereby demonstrating the importance of considering the network context when inferring metabolic changes from transcriptomic data. Overall, our approach has revealed two major metabolic differences at the level of LCFA utilization with relevance for tumor proliferation, invasiveness and metastasis in our dual cell model.

First, it has unveiled an increased CPT1 activity in PC-3/M cells. CPT1 has numerous cytosolic substrates, including cervonyl coenzyme A (DHA precursor), eicosatetranoyl coenzyme A, arachidyl coenzyme A (arachidonic acid precursor), trans-2-octadecenoyl-CoA(4-), palmitate, Malonyl-CoA, linoelaidyl coenzyme A (linoleic acid precursor) and vaccenyl coenzyme A. Interestingly, it has been reported that five of these eight LCFAs have antiproliferative effects [16–20]. Thus, the higher CPT1 and β-oxidation activities in PC-3/M cells may have two roles: i) first, and probably the most evident, is to maintain the energetic requirements imposed by the high proliferation rates of PC-3/M cells and ii) to eliminate LCFAs with antiproliferative effects. The predicted differences in CPT1 activity were supported experimentally by the finding of 33% higher CPT1 protein levels in PC-3/M than PC-3/S cells. Further, we demonstrated that β-oxidation activity was more sensitive to the inhibition of CPT1with etomoxir in PC-3/M than in PC-3/S cells (see Fig 2B) which highlights the importance of this enzyme in the energy metabolism of this subpopulation. This is of special interest since fatty acid oxidation plays a key role as source of NADH, NADPH, ATP and FADH2, all providing survival advantage to cancer cells [41]. Finally, we showed that the concentration of DHA is significantly higher in PC-3/S than in PC-3/M cells (Fig 2D). Several studies have reported anti-proliferative effects of DHA in tumors, consistent with the high proliferative rate observed in PC-3/M cells and support the hypothesis that an increased activity of CPT1 is also necessary to eliminate anti-proliferative molecules in PC-3/M cells. Taken together, our findings supports the key role played by CPT1 to sustain the high proliferation rate of PC-3/M cells by degrading LCFAs through energy metabolism while avoiding their antiproliferative effects.

Secondly, our analysis predicted a higher activity of the eicosanoid metabolism in PC-3/S cells. Most of the LCFAs described in this study are AA precursors that in turn fuel this pathway. Eicosanoid metabolism produces a variety of molecules with reported tumorogenic activity in prostate cancer [42]. Importantly, these processes are predicted to occur in the lysosome, in which is reported that may produce pro-oncogenic alterations [43]. Here we validated this prediction by using metabolomic measurements which revealed higher levels of AA in PC-3/S (see methods and Fig 3). In addition, in line with the computational predictions, the concentrations of several products of eicosanoid metabolism, such as 12S-HETEand PGE2, were significantly higher in the PC-3/S subpopulation. Prior evidence suggests that these compounds are associated with the upregulation of *ITGA3V*, *VEGF* and *AKT* [21–23, 39], which promote cell adhesion, angiogenesis and cell invasion. Importantly, we have found that these genes are indeed upregulated in PC-3/S cells. Thus, our findings suggest that higher levels of AA, 12S-HETE and PGE2, associated with a more active eicosanoid metabolism in PC-3/S cells, contribute to increased angiogenesis, cell adhesion and invasion potentials of these cells.

**Fig 4:**
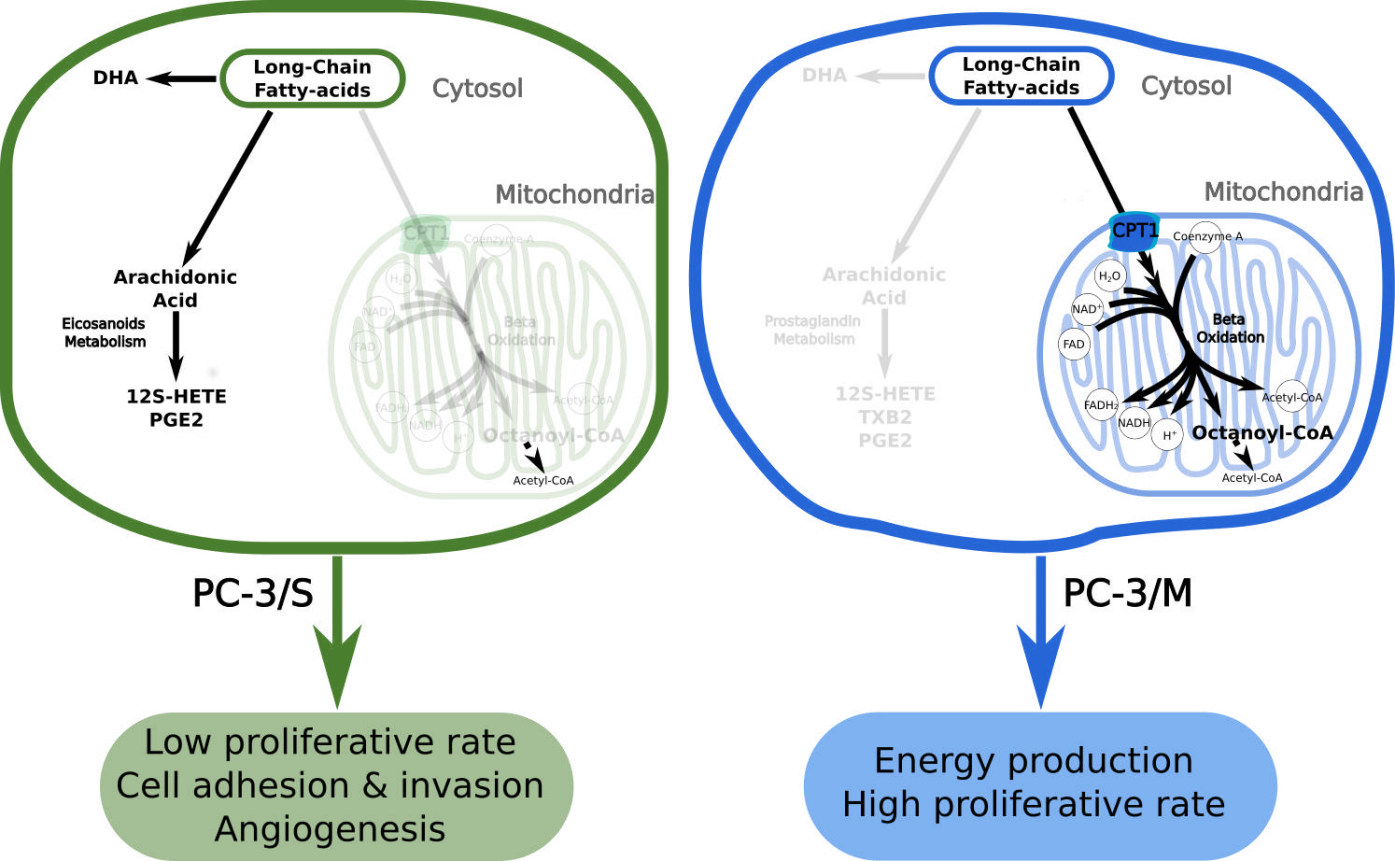
Proposed mechanism of metabolic reprogramming and the resultant phenotypes associated with PC-3/M and PC-3/S cells. A: Metabolic pathways predominantly active in PC-3/S cells (black solid arrows). Inactive/poorly active pathways are represented as blurred arrows. B: Metabolic pathways predominantly active in PC-3/M cells (black solid arrows). Inactive/poorly active pathways are represented as blurred arrows.

Overall, our findings support the view that the relatively high activity of CPT1 in PC-3/M cells increases the entry of LCFAs into the mitochondria to be oxidized and to produce energy to sustain a high proliferation rate (Fig 4). This process decreases the levels of LCFAs such as DHA, thus preventing their antiproliferative effects. In contrast, the lower CPT1 activity in PC-3/S cells would lead to an accumulation of anti-proliferative LCFAs in the cytosol, thereby reducing the growth rate in PC-3/S cells and increasing the availability of substrates for eicosanoid synthesis (Fig 4A). Summing up, we propose that the metabolic reprogramming involving LCFA utilization enhances the metastatic potential and proliferation in PC-3/M cells while in PC-3/S subpopulation increases cell adhesion, invasion and angiogenic capability and promotes DHA accumulation which reduces its proliferation.

The model-driven analysis employed here has provided additional insights into metabolic changes linked to cancer phenotypes. For example, our analysis further predicted acid ceramidase (ASAH1) to be active predominantly in PC-3/M cells, a prediction consistent with experimental evidences showing a higher ASAH1 enzymatic activity in this subpopulation [44]. Our analysis also predicts that calcitriol metabolism is mainly active in PC-3/S cells. This molecule has antiproliferative activity in a variety of human cancer cells [25] which is consistent with the low proliferative rate of PC-3/S cells compared with PC-3/M cells [9]. Our model prediction also suggests that the reported low sensitivity of PC-3 cells towards Vitamin D3 [24] could be conferred by the low Vitamin D metabolism activity in the PC-3/M subpopulation. In addition, this prediction is consistent with the lower proliferative rate observed in PC-3/S cells, which would be more sensitive to Vitamin D3 anti-proliferative effects.

Our analysis of a dual-cell model representing distinct and opposing neoplastic phenotypes allows us to propose subpopulation-specific and complementary therapeutic interventions. The results of the experiment determining the dose-effect relation between etomoxir concentration and cell proliferation showed that PC-3/M cells are more sensitive to etomoxir than PC-3/S cells, and that the parental PC-3 cell line presents an intermediate sensitivity. Thus, the poor performance of etomoxir at inhibiting the growth of PC-3 cells compared to other prostate cell lines may be explained by the low metabolic dependence of the PC-3/S subpopulation on CPT1. In other words, androgen-independent prostate cancer cells with CSC attributes similar to PC-3/M cells would be sensitive to etomoxir, while this drug would be less efficient in tumor cell subpopulations with EMT attributes similar to PC-3/S cells with a phenotype characterized by high cell invasion and adhesion and angiogenic capability.

Finally, it has been reported that the cox-2 reaction, which produces PGE2 and is over-expressed in prostate cancer [45], is activated by 12S-HETE [46] which is in turn metabolized by the 12-LOX reaction. Interestingly, a number of drugs such as cinnamyl-3,4-dihydroxy-alpha-cyanocinnamate (CDC) or baicalein, that inhibit 12-LOX activity, have been shown to present strong anti-tumoral effects in prostate cancer [47].

Our study represents a novel approach to discern metabolic vulnerabilities associated with heterogeneous tumor cell populations. However, future studies measuring the effects of single and combinatorial drug treatments affecting subpopulation-specific targets on heterogeneous co-culture of non-CSC (PC-3/S) and CSC (PC-3/M) subpopulations are needed to determine the significance of these findings. For instance, the combinatorial effect of CDC or baicalein with drugs such as oxfenicine or perhexiline (CPT1 inhibitors without the hepatotoxicity of etomoxir–[48]) could be tested as potential anti-tumoral drug treatments targeting the key metabolic processes preferentially active in PC-3/S or PC-3/M cells, respectively. Additionally, as gene networks associated with progression and metastasis in our PC-3 dual model is significantly correlated with those in other tumor types [14], the metabolic reprogramming proposed here could be extrapolated to different cancer types. Our findings will facilitate a better understanding of the EMT-induced metabolic changes and their role in tumor heterogeneity and opens new avenues for the development of new subpopulation-specific anti-cancer therapies.

## Methods

### Experimental data

Transcriptomic data: Gene expression levels of each cell subpopulation (three replicates per subpopulation GSE24868, [9]) by microarray analysis (*Affymetrix* genechip u133a 2.0) and normalized by RMA [49]. Transcriptomic data was integrated into a genome-scale metabolic network reconstruction analysis to infer the activity state of the metabolic reactions in both subpopulations.

Consumption and production of metabolites: Additionally, we used the measured consumption and production of some metabolites [14] to assess the reliability of model predictions (Supplementary information S1 Text). These metabolites were: glucose, lactate, pyruvate, glutamate and aminoacids.

### Metabolic model

To obtain accurate cell-specific genome-scale metabolic models of the PC-3 subpopulations, we performed a subpopulation-specific genome-scale network reconstruction analysis by integrating the transcriptomic data into the most recent reconstruction of human metabolism (Recon2) [11]. Recon2 is a genome-scale stoichiometric model that represents the entire network of human metabolic reactions. This generic genome-scale metabolic model provides the appropriate transcript-protein-reaction associations that permit the integration of the previously mentioned transcriptomic data for which we used a widely tested constraint-based method [12].

### Model reduction

In order to reduce the computational time necessary to perform the analysis, the metabolic model (Recon2) [11] was reduced. The reduction was done by removing the blocked reactions from the model. These reactions are those incapable of carrying any metabolic flux in steady state [50]. To this aim we first performed a Flux Variability Analysis (FVA) [51–53] using Fasimu software [54]. This analysis computes minimal and maximal flux in each reaction. Each analysis evaluates the feasibility of the simulation. The reactions in which their maximization and minimization simulations were not obtained any feasible solution were considered as blocked reactions. In order to ensure that the reduced model was able to consume/produce the experimentally measured extracellular metabolites, we forced the corresponding exchange reactions to be always active. It was achieved by splitting all the exchange reactions in a forward and a backward reaction and the lower/upper bounds of the reactions associated to the experimentally measured metabolites were fixed at 0.001/1000 in the forward reactions and at -1000/-0.001 in the backward reactions. Once determined, the blocked reactions were removed from the model, as well as those metabolites that were neither products nor substrates of any reaction.

### Transcriptomic data integration

We integrated the transcriptomic data into Recon2 [11] by using the gene-protein-reaction (GPR) associations included in the model. These associations are “and/or” logical sentences that establish a relation between the metabolic reactions and the genes encoding the enzymes that catalyze them. GPR associations include information related with isoenzymes (using the logical “or”), complexes (using the logical “and”) or direct gene-reaction relations (i.e. the activity of Reaction1 depends on: “(geneA and geneB) or (geneC and geneD)”). To integrate the gene expression data from PC-3/M and PC-3/S subpopulations into Recon2 and determine the gene expression level associated to the metabolic reactions in each subpopulation, we substituted the logical “and” and “or” by “minimum” and “maximum”. Thus, for example, if the activity of a given reaction depends on the expression of different genes and it is defined by the following logical expression “(geneA and geneB) or (geneC and geneD)”, and the expression of the gene A, B, C and D are 0.5, 3, 1 and 0.1 respectively. Then, by integrating the gene expression levels into the logical sentence and replacing the logical operators by “minimum” and “maximum” we obtained the following expression: “max(min(0.5,3),min(1,0.1))”. Thus, based on the transcriptomic data and the GPR association, the gene expression associated with the reaction is 0.5. Finally, we obtained a numerical value for each reaction indicating the level of expression of their corresponding associated genes.

### Expression-based activity prediction

We used gene expression levels associated with the metabolic reactions to infer the activity states of reactions in the network by using a recently developed constraint-based method [12]. This method solves a mixed integer linear programming (MILP) problem to obtain a flux distribution in which the number of reactions associated with highly expressed genes is maximized (R_H_), and the number of reactions associated with lowly expressed genes is minimized (R_L_) while satisfying the thermodynamic and stoichiometric constrains imposed by the model:

$${max}_{v,y^{+},y^{-}}=(\sum_{i\in R_{H}}(y_{i}^{+}+y_{i}^{-})+\sum_{i\in R_{L}}y_{i}^{+})$$

S∈v=0 (1)

The mass balance constraint: where v is the flux vector and S is a n x m stoichiometric matrix, in which n is the number of metabolites and m is the number of reactions.

$$v_{min}\leq v\leq v_{max}$$ (2)

Thermodynamic constraints, that restrict flow direction, are imposed by setting v_min_ and v_max_ as lower and upper bounds respectively.

$$y_{i}^{+},y_{i}^{-}[0,1]$$ (3)

The Boolean variables y+ and y–. In R_H_ reactions represent whether the reaction is active or not respectively. In R_L_ y+ represents the reaction is not active.

$$v_{i}+y_{i}^{+}(v_{min,i}-\varepsilon )\geq v_{min,i},i\in R_{H}$$ (4)

A highly expressed reaction is considered to be active if it carries a significant positive flux that is greater than a positive threshold Ɛ. In our study Ɛ = 1. Consequently the ith reaction is active if: *v*_*i*_ ≥ 1

$$v_{i}+y_{i}^{-}(v_{min,i}+\varepsilon )\leq v_{max,i},i\in R_{H}$$

or has a significant negative flux <–Ɛ (as our model didn't consider reversible reactions it cannot occur)

$$v_{min}(1-y_{i}^{+})\leq v_{i}\leq v_{max,i}(1-y_{i}^{+}),i\in R_{L}$$ (5)

**Fig 5:**
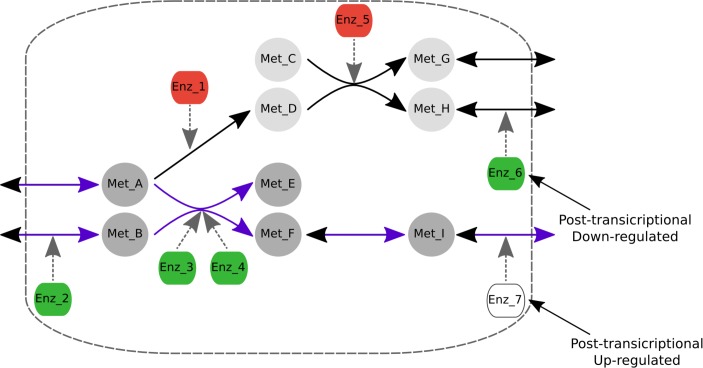
Transcriptomic-based algorithm on a toy metabolic network. Here the nodes from Met_A to Met_I represents the metabolites involved in this network, the metabolic reactions are represented by continuous arrows, nodes Enz_1 to Enz_7 represent the enzymes that catalyze the metabolic reactions and the discontinuous arrows indicate the enzyme to which each metabolic reaction is associated. The enzymes associated with highly expressed genes are highlighted in green, those associated with lowly expressed genes in red and enzymes associated with moderately expressed genes in white. The algorithm penalizes the use of reactions associated with lowly expressed genes and rewards the use of those associated with highly expressed genes. Thus, based on the expression of the genes associated with this metabolic network, the algorithm predicts that the reactions highlighted in purple will be active while the reactions in black will be inactive. Consequently the metabolite Met_I will be secreted but not Met_G or Met_H.

Lowly expressed reactions are considered to be inactive if they carry zero metabolic flux, though changing Eq (5) to enable these reactions to carry a low metabolite flux (that is, with an upper bound lower than Ɛ) and still be considered inactive provides qualitatively similar results. The Fig 5 illustrates the process.

This method defines an upper threshold above which the expression of a given gene is considered high and another threshold below which gene expression is considered low. In our study, the chosen upper and lower thresholds were those symmetric percentiles that maximize the cases where the number of reactions associated with highly expressed genes in one subpopulation were associated with lowly expressed gene in the other subpopulation and vice versa. Thus, we defined the upper threshold at the 66^th^ percentile and the lower threshold at the 33^th^ percentile. The method also uses the parameter that represents the flux above which a given reaction is considered to carry a significant metabolic flux. As is defined in [12] we gave to Ɛ a value of 1. Once the thresholds were fixed, we performed the expression-based activity prediction analysis with Fasimu software by applying “compute-FBA–xs” option (See Fasimu tutorial [54]).

### Sensitivity analysis

In the Expression-based activity prediction analysis we found an optimal solution in terms of the objective function maximization, although this solution may not be unique. A space may exist of alternative optimal solutions that represent alternative steady-state flux distributions yielding the same similarity with the gene expression data (the same objective function value).

To account for these alternative solutions, we employed Sensitivity analysis [12].

This is performed by solving two MILP problems (as is described in Expression-based activity prediction) for each reaction to find the maximal attainable similarity with the expression data when the reaction is: (i) forced to be activated and (ii) forced to be inactivated.

Thus, a reaction is considered to be active if a higher similarity with the expression data is achieved when the reaction is forced active than when it is inactive (the objective function is higher when the reaction is active). Conversely, it is considered to be inactive if the similarity is higher when the reaction is forced to be inactive. If the similarities with the experimental data are equal in both cases the activity state of the reaction is considered to be undetermined. From this analysis we could infer which pathways are more active in each subpopulation.

### Reliability of model predictions

By analyzing the predicted activity state of the exchange reaction we can infer which metabolites are consumed and/or produced. In order to determine the goodness of our model predictions we compared qualitatively the consumption and production of some experimentally measured metabolites [14] with the corresponding model predictions (Supplementary information S1 Text). This comparison was done by constructing a 2x2 contingency matrix and the levels of significance were determined using Fisher exact test (Supplementary information S1 Text).

### Robustness analysis

The algorithm used to integrate the information from gene expression levels into a Genome-scale metabolic network reconstruction defines a threshold above which gene expression levels are considered high and a second threshold below which they are considered low. It calls for the performance of a robustness analysis in order to demonstrate the lack of dependency of our predictions on the thresholds used in the analysis. In order to determine the robustness of our prediction, we performed the analysis previously described in sensitivity analysis defining different sets of thresholds:

- upper threshold: 25^th^ percentile; lower threshold: 75^th^ percentile
- upper threshold: 33^th^ percentile; lower threshold: 66^th^ percentile
- upper threshold: 40^th^ percentile; lower threshold: 60^th^ percentile

Thereby, we defined a set of reactions that were predicted to be active, inactive or undetermined (the method cannot predict their activity state) independently of the thresholds.

### Oxygen consumption rate (OCR)

Cells were seeded in XF24-well microplates (Seahorse Bioscience) at 4.5·10^4^ cells/well and 9.0·10^4^ cells/well, respectively, in 100 μL of growth medium, adding 100 μL more after 3–5 h, and then incubated at 37°C with 5% CO_2_ overnight. After overnight incubation and 1 h before the assay, growth media was replaced by basal media (unbuffered DMEM; Sigma-Aldrich) with 3 mM glucose and 5 mM carnitine. The sensor cartridge was loaded with etomoxir and calibrated prior to the start of the experiment. Determinations were performed on a XF24 Extracellular Flux Analyzer (Seahorse Bioscience). Responses to etomoxir (Signma-Aldrich) treatment (final concentration 30 μM) were expressed as LOG2 to indicate the fold change comparing the measured point immediately after and before the corresponding injection.

### Eicosanoid and other oxidation products of polyunsaturated fatty acids (PUFAs)

To determine eicosanoids and oxidation products of polyunsaturate fatty acids levels in PC-3/M and PC-3/S cells we used Biocrates triple quadrupole MS-based platforms [32]. This platform enables the systematic quantification of relevant biological metabolites. The method is a quantitative screen of selected metabolites detected with multiple reaction monitoring, neutral loss and precursor ion scans. Metabolites are then quantified by comparison to structurally similar molecules labeled with stable isotopes added to the samples in defined concentrations as internal standards. The process is controlled by MetIDQ Software which controls sample management, data collection, data validation, and analysis.

### Western blotting

Cell extracts were obtained from frozen cell pellets using RIPA buffer (50 mM Tris,pH 8.0, 150 mM NaCl, 0.1% SDS, 1% Triton X-100 and 0.5% sodium deoxycholate) supplemented with protease inhibitor cocktail (Sigma-Aldrich). Protein concentrations from the supernatant were determined by the BCA assay. Thirty-five mg of protein per sample were loaded and separated by 10% SDS-PAGE and transferred to PVDF membranes. Membranes were blocked by incubation with PBS-Tween (0.1% (v/v)) containing 5% non-fat dried milk for 1 hour at room temperature. Then, membranes were incubated with CPT1 primary antibody (Sigma-Aldrich, SAB1410234, 1/200), rinsed with PBS-Tween (0.1% (v/v)) and finally incubated with the secondary antibody anti-rabbit (Amersham Biosciences, NA934V, 1/3000) for 1 hour at room temperature. Blots were treated with the Immobilon ECL Western Blotting Detection Kit Reagent (Millipore) and developed after exposure to Fujifilm X-ray film.

### Acylcarnitines measurement

For sample acquisition and processing, 5 10^6^ cells of PC-3M and PC-3S cell lines were tripsinized and washed twice with ice-cold PBS prior to snap-freezing in liquid nitrogen. Cell pellets were stored at -80°C until measure. Right before measuring, cell pellets were thawed at room temperature and resuspended in 70 μL of 85:15 EtOH:PBS solution. Cells were disrupted by two sonication/freezing/defreezing cycles using titanium probe (VibraCell, Sonics & Materials Inc., Tune: 50, Output: 25), liquid N2 and a 95°C heat block. Cell lysates were then centrifuged at 20,000 rcf for 5 minutes at 4°C. Supernatants were collected into new tubes and total protein content was determined by Bichinconinic acid (BCA) assay (Thermo Fisher Scientific, Waltham, MA USA).

Then, standards, internal standards, quality controls (10 μL of each), and samples (30 μL) were loaded into the Biocrates AbsoluteIDQ® p180 plates (BIOCRATES Life Sciences AG, Innsbruck, Austria), processed according to manufacturer instructions and measured by FIA-MS/MS using a SCIEX 4000 QTRAP mass spectrometer.

Concentrations for metabolites were determined using the MetIDQ™ software package, which is an integral part of the AbsoluteIDQ® kit. The obtained metabolite concentrations were corrected considering the loaded volume of cell lysates and normalized by protein content.

## Supporting information

S1 File Metabolic reaction activity state. (XLS)

S2 File Pathway activity state. (XLS)

S3 File List of reactions active only in one subpopulation predicted by the robustness analysis. (XLS)

S4 File Eicosanoid and PUFAs levels in PC-3/M and PC-3/S cells. (XLS)

S5 File Dose-effect relation between etomoxir concentration and cell proliferation in PC-3, PC-3/M and PC-3/S. (XLS)

S6 File Acylcarnitine levels in PC-3/M and PC-3/S cells. (XLS)

S1 Text Experimental validation of metabolic consumptions and production and pathway analysis. (PDF)
