# Supplementary material for: Model-driven discovery of long-chain fatty acid metabolic reprogramming in heterogeneous prostate cancer cells
Source: PLoS Comput Biol. 2018 Jan 2;14(1):e1005914. doi: 10.1371/journal.pcbi.1005914 (PMC5766231; doi:10.1371/journal.pcbi.1005914)
Supplement: S1 Text — (PDF) [file pcbi.1005914.s007.pdf]

i) Predicted consumptions and productions

PC-3/M		PC-3/S	
Consumed	Produced	Consumed	Produced
Sodium	**L-cysteinylglycine**	Sodium	L-cysteinylglycine
**L-alanine**	5-L-Glutamyl-L-alanine	**L-alanine**	5-L-Glutamyl-L-alanine
**L-argininium**	keratan sulfate II (core 2-linked)	**L-argininium**	keratan sulfate II (core 2-linked)
**L-asparagine**	keratan sulfate I	**L-asparagine**	keratan sulfate I
5-L-Glutamyl-L-alanine	PA6	5-L-Glutamyl-L-alanine	N-Acetyl-beta-D-glucosaminyl-1,2-alpha-D
**L-glutamine**	Taurine	**L-glutamine**	**L-methionine**
**Glycine**	2-Hydroxybutyrate	**Glycine**	**L-threonine**
**L-histidine**	proton	**L-histidine**	**L-cysteine**
L-homoserine	acetoacetate	L-homoserine	cholesterol ester
keratan sulfate II (core 2-linked)	acetone	keratan sulfate II (core 2-linked)	Taurine
keratan sulfate I	Biotin	keratan sulfate I	2-Hydroxybutyrate
**L-leucine**	butyrate	**L-leucine**	proton
**L-lysinium**	Sodium	**L-lysinium**	3,4-Dihydroxyphenylethyleneglycol
**L-methionine**	creatine	**L-methionine**	acetoacetate
Ornithine	**R-lactate**	Ornithine	acetaldehyde
**L-phenylalanine**	Bicarbonate	**L-phenylalanine**	acetone
**L-proline**	**S-lactate**	**L-proline**	alpha-pinene
PA6	methanol	**L-serine**	Biotin
**L-serine**	**pyruvate**	**L-threonine**	butyrate
**L-threonine**	trans-vaccenate	Albumin	carbon monoxide
Bicarbonate	Albumin	taurolithocholate	coumarin
Chloride	Flavin adenine dinucleotide oxidized	sulfochenodeoxycholate	cortisol
Taurine	**L-alanine**	Bicarbonate	glycochenodeoxycholic acid
**L-tryptophan**	**L-cysteine**	Chloride	**R-lactate**
caprate	**L-asparagine**	Taurine	2,6 dimethylheptanoyl carnitine
2-Hydroxybutyrate	**L-threonine**	**L-tryptophan**	Bicarbonate
proton	**L-lysinium**	**L-tyrosine**	Sodium
acetoacetate	**L-argininium**	**L-cysteine**	Hexadecanoate (n-C16:0)
acetone	Ornithine	**L-valine**	globoside
Biotin	S-glutathionyl-2-4-dinitrobenzene	**L-isoleucine**	GD1c
butyrate	S-glutathionyl-ethacrynic-acid	caprate	**D-glucose**
creatine	glutathionyl-leuc4	laurate	cerotate
**R-lactate**	glutathionate	Deoxyinosine	ammonium
**S-lactate**	hydrogenphosphate	2-Hydroxybutyrate	Chloride
methanol		proton	potassium
**pyruvate**		3,4-Dihydroxyphenylethyleneglycol	leukotriene D4
Albumin		acetoacetate	**S-lactate**
Flavin adenine dinucleotide oxidized		acetaldehyde	octadecanoate (n-C18:0)
**L-cysteine**		acetone	octadecenoate (n-C18:1)
S-glutathionyl-2-4-dinitrobenzene		alpha-pinene	**pyruvate**
S-glutathionyl-ethacrynic-acid		Biotin	D-ribose
glutathionyl-leuc4		butyrate	taurochenodeoxycholic acid
glutathionate		clupanodonic acid	tetracosahexaenoic acid
hydrogenphosphate		carbon monoxide	tetracosapentaenoic acid
		coumarin	Albumin
		creatine	Flavin adenine dinucleotide oxidized
		cortisol	UTP
		hydrogen cyanide	Riboflavin
		glycochenodeoxycholic acid	**L-glutamine**
		**R-lactate**	**L-alanine**
		2,6 dimethylheptanoyl carnitine	**L-serine**
		globoside	glutathionyl-leuc4
		GD1c	S-glutathionyl-2-4-dinitrobenzene
		**D-glucose**	S-glutathionyl-ethacrynic-acid
		Hexadecenoate (n-C16:1)	glutathionate
		cerotate	prostaglandin-b1
		myo-inositol	prostaglandin-b2
		ammonium	prostaglandin-d1
		potassium	prostaglandin-f2beta
		leukotriene B4	hydrogenphosphate
		leukotriene D4	
		**S-lactate**	
		octadecenoate (n-C18:1)	
		**pyruvate**	
		D-ribose	
		tetracosahexaenoic acid	
		tetracosapentaenoic acid	
		cholesterol ester	
		Flavin adenine dinucleotide oxidized	
		Riboflavin	
		glutathionyl-leuc4	
		S-glutathionyl-ethacrynic-acid	
		S-glutathionyl-2-4-dinitrobenzene	
		glutathionate	
		leukotriene C4	
		prostaglandin D2	
		hydrogenphosphate	
		**D-aspartate**	

Table 1: Predicted consumption and productions. This table shows those metabolites that are predicted to be consumed and produced in PC-3/M (first and second column respectively) and PC-3/S subpopulations (thirst and fourth column respectively). The metabolites highlighted in bold letter were compared with experimental measurements.

ii) Evaluate the reliability of model predictions

By analyzing the activity state of the reactions that exchanges metabolites between the cell and the media (exchange reactions) we determined which metabolites were predicted to be consumed or produced by PC-3/M and PC-3/S cells . We compared the predicted consumptions and productions with experimental measurement (Aguilar *et al.* 2016). The following table summarizes this comparison:

	$PC-3M$		$PC-3S$	
Metabolites	*Consumption*	*Production*	*Consumption*	*Production*
Glucose	✗	✓	✓	✗
Glutamine	✓	✓	✓	✗
Threonine	✓	✗	✓	✗
Serine	✓	✓	✓	✗
Arginine	✓	✗	✓	✓
Proline	✗	✗	✓	✓
Valine	✗	✓	✓	✓
Cysteine	✓	✗	✓	✗
Methionine	✓	✓	✓	✗
Isoleucine	✗	✓	✓	✓
Leucine	✓	✓	✓	✓
Tyrosine	✗	✓	✓	✓
Phenylalanine	✓	✓	✓	✓
Lysine	✓	✗	✓	✓
Histidine	✓	✓	✓	✓
Tryptophan	✓	✓	✓	✓
Glycine	✓	✓	✗	✗
Lactate	✗	✓	✗	✓
Asparagine	✓	✗	✓	✓
Alanine	✗	✓	✗	✓
Pyruvate	✓	✗	✓	✗
Aspartate	✗	✓	✓	✗

Table 2: The metabolites that were evaluated are in the first column. Red crosses represent wrong predictions and green check represent right predictions. The comparisons of consumption and production of PC-3/M cells are in the second and third column respectively. The comparisons of consumption and production in PC-3/S cells are in the fourth and fifth column respectively.

This table shows those metabolites that are predicted to be consumed and produced in PC-3/M (first and second column respectively) and PC-3/S subpopulations (thirst and fourth column respectively). The metabolites highlighted in bold letter were compared with experimental measurements.

PC-3/M	Y-experimental	N-experimental
Y-predicted	17	10
N-predicted	6	13
	65,2173913043	

SUMM	Y-experimental	N-experimental
Y-predicted	38	21
N-predicted	8	25
	68,4782608696	

PC-3/S	Y-experimental	N-experimental
Y-predicted	21	11
N-predicted	2	12
	71,7391304348	

Table 3: Table of contingencies

The table of contingencies above summarizes the comparison between the measured consumption and production of metabolites and their corresponding model predictions. In table A are the results corresponding to PC-3/M cells, in table C the results corresponding to PC-3/S and the table B summarizes both subpopulations. The diagonal of gray squares represents those predictions of consumption or production supported by experimental observation (true positives in the coordinates Y-experimental,Y-predicted) and those cases where the model predict that a metabolite is not produced or consumed and is supported by experimental observations too (true negatives in the coordinates N-experimental,N-predicted). The white diagonal corresponds to those predictions that are not consistent with the experimental observations. The white square at the coordinate Y-predicted, N-experimental summarizes the predictions of consumption and production not supported by the experimental observations (false positives) and the white square in the coordinate N-predicted,Y-experimental corresponds to those cases where a certain metabolite is observe to be produced or consumed and is not predicted by the model (false negative). Finally the gray square bellow each table of contingencies represents the % of right predictions. Thus, the model was able to predict correctly the 65.22% and the 71.74% of consumptions and productions in PC-3/M and PC-3/S respectively, and the 68,48% if we consider both subpopulations. Next we performed a fisher exact test to determine the corrected 2 tailed p-value. Thus, the p-value associated to the overall predictions is 0,0003 indicating a high level of confidence of our model predictions.

iii) Metabolic pathways and biological functions differentially active

Each reaction in the model is associated to a certain pathway (subsystem in the model). Based on this association and the results obtained in the previous analysis, we determined the activity of the pathways in each subpopulation. It is summarized in the following graph:

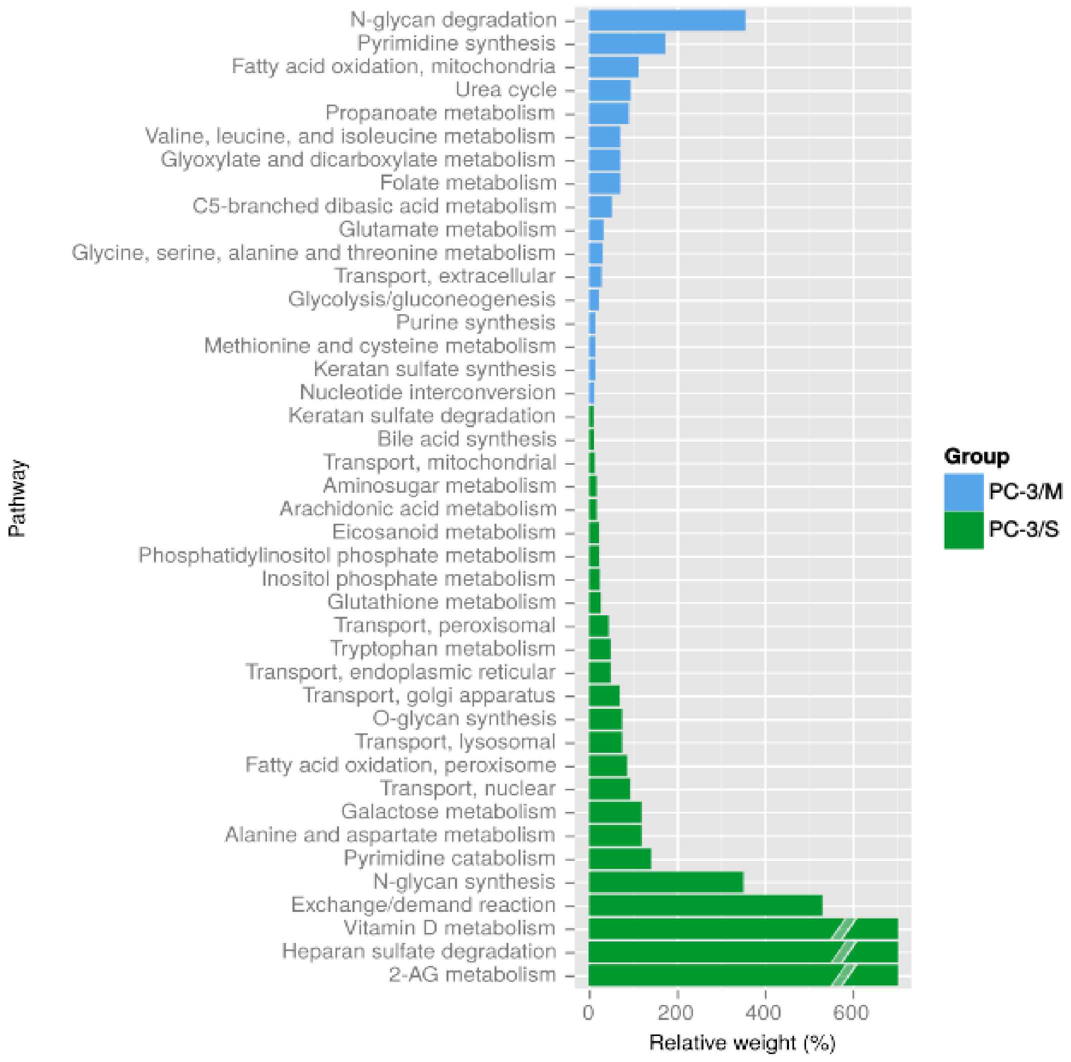

Figure 1. Pathways relative weight. In this figure are represented those metabolic pathways with an activity state 10% higher in one of the subpopulations and a corrected p-value lower than 0.05 and the pathways that are active only in one of the subpopulations. Blue bars represent the % of over activity in pathways that are more active in PC-3/M and green bars represent the pathways with more metabolic activity in PC-3/S. The over-activity of those metabolic pathways that are active only in one of the subpopulations.

We first asked which metabolic pathways differ most in their activity state between the two subpopulations (Figure 1, and Supplementary material 2). Most notably, the analysis revealed a strong pattern of complementarity in the predicted pathway activity state between the two subpopulations. For example, N-Glycan degradation pathway more active in PC-3/M than in PC-3/S cells, while, the activity of O-Glycans and N-glycans synthesis pathways are more active in the PC-3/S subpopulation. As alterations in glycan metabolism has been reported to enhance the metastatic capability of PC-3 cells to bone (Peracaula R *et al.* 2003), the predicted metabolic reprogramming in N-glycans metabolism may play an important role in cancer progression and metastasis. In a similar vein, the computational analysis predicts that Purines and Pyrimidines are

mainly synthesized in PC-3/M cells while their catabolism is more active in PC-3/S subpopulation. Glutamate metabolism and Urea cycle are also more active in PC-3/M cells, this processes provide amino groups for the "de novo" synthesis of purine and pyrimidine bases (Bardot V *et al.* 1994) that are necessary for the replication of DNA. These profiles fit with the more proliferative phenotype of PC-3/M cells (Brézillon S *et al.* 2013). We also found several differences in fatty acid and lipid metabolism. Vitamin D3 metabolism is also predicted to be exclusively active in PC-3/S subpopulation. This molecule controls the proliferation in prostate cells (Munetsuna E *et al.* 2014) and has antiproliferative effects on a number of cancer cell types (Trump DL et al. 2006). This prediction is consistent with the lower proliferative ration observed in PC-3/S cells.

iv) Evaluate the accuracy of pathway activity predictions

In order to determine the accuracy and the levels of confidence of the pathway activity predictions we performed an statistical analysis based on bootstrap methodology (Wang C *et al.* 2016). The algorithm that was developed for this aim is summarized in the figure bellow:

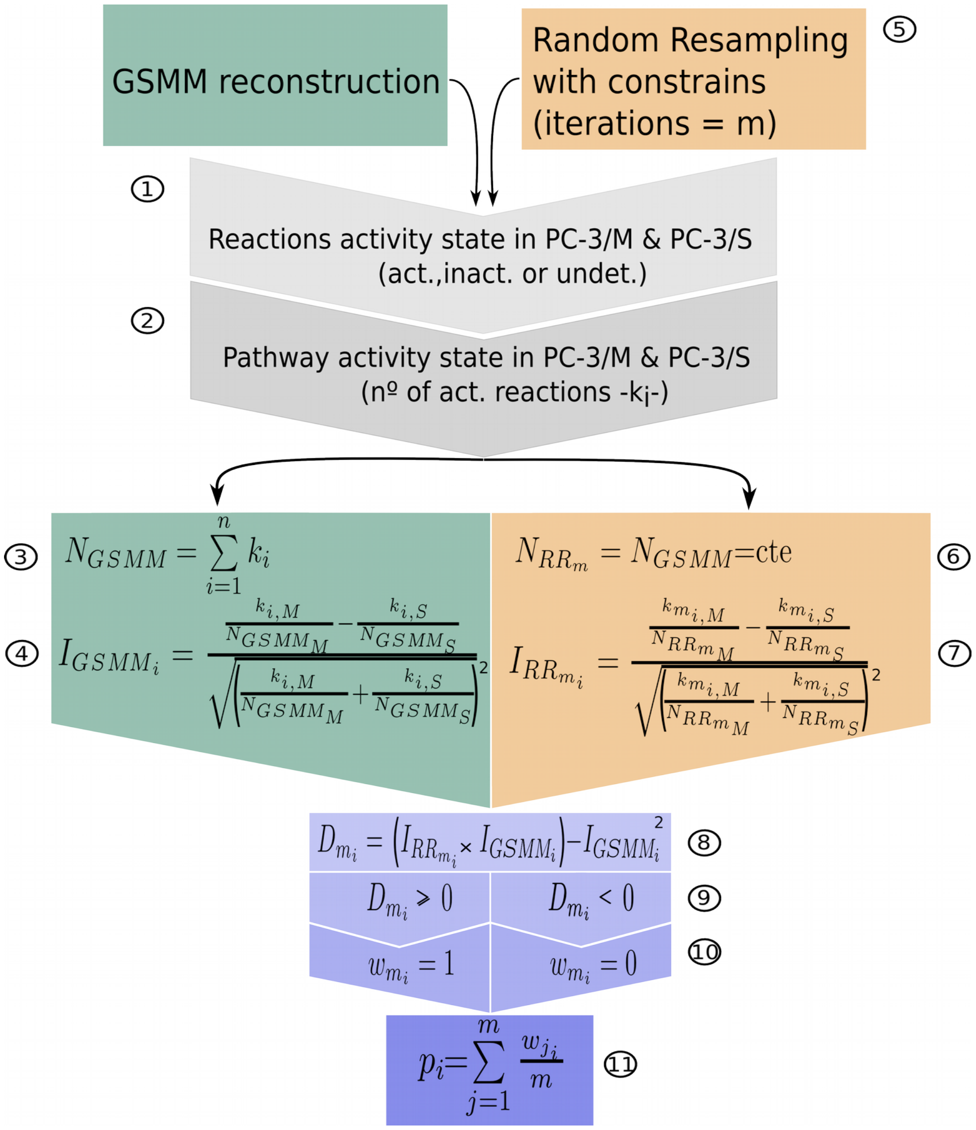

Figure 2: This figure represents method used to determine the accuracy of the pathways activity predictions

This method can be divided in three main parts: i) define the statistics of the GSMM reconstruction analysis (I_{GSMM}) , ii) define the statistics of the random re-sampling analysis (I_{RRMi}) and iii) determine the level of significance of model prediction by comparing I_{GSMM} and I_{RRMi}.

We start by determining the statistical parameters of the GSMM reconstruction analysis. First we determine the activity state of the metabolic reactions in PC-3/M and PC-3/S subpopulations as is described previously in the sensitivity analysis section (1). Secondly, is calculated the activity state of the metabolic pathways by grouping the active metabolic reactions based on their associated pathways (2). Next is determined the total number of active reactions in each subpopulation (N_{GSMM}) (3) by summing all the active reactions. Finally we define the statistic I (4) that is defined by the following symmetric function:

$$I_i = \frac{\frac{k_{i,M}}{N_M} - \frac{k_{i,S}}{N_S}}{\sqrt{\left(\frac{k_{i,M}}{N_M} + \frac{k_{i,S}}{N_S}\right)^2}} \quad \text{eq 1}$$

This statistic (I_{GSMM}) provides a numerical value defining the differential activity state between PC-3/M and PC-3/S in the ith pathway and can have values between -1 (pathway active only in PC-3/S) and 1 (pathway active only in PC-3/M), when it is 0 the activity in the ith pathway is equally active in both subpopulations.

Once we have defined the parameters related with the GSMM reconstruction analysis, we performed a random re-sampling analysis based on bootstrap methods (Wang C *et al.* 2016) (5). Briefly, this method generates a random distribution of the active reactions within the pathways defined in the model satisfying that the total number of active reactions must be equal to N_{GSMM} (6). Next is defined the statistic I for the ith pathway in the mth iteration (I_{RRMi}) (7).

Finally, are compare the statistic I_{GSMM}, defined from the results obtained in the GSMM analysis and the statistic I_{RRmi} calculated from the mth random re-sampling by using the following expression(8):

$$D_{m_i} = \left(I_{RR_{m_i}} \times I_{GEMN_i}\right) - I_{GEMN_i}^{2} \qquad \text{eq 2}$$

If D_{mi} is equal or higher than 0 the relation between subpopulations of the activity state of the ith pathway in the mth iteration is equal or more extreme than the activity state calculated in the GEMN analysis otherwise D_{mi} is lower than 0 (9). Note that when the activity state of both subpopulations is equal, I_{GSMM} is 0 and consequently D_{mi} is always 0. In order to avoid this limitation we first ensured that there were no pathways with the same activity state between PC-3/M and PC-3/S. Next, we define the boolean variable w ($w \in \{0,1\}$), then if D_{mi} is 0 w_{mi} is 1 and if D_{mi} is different to 0 w_{mi} is 0 (10). Finally, the statistic p, that evaluates the accuracy of pathway activity state predicted by the model is calculated by using the following equation (eq 3):

$$p_i = \sum_{j=1}^{m} \frac{w_{j_i}}{m} \qquad \text{eq 3}$$

In this analysis the number of iterations (m) was 10.000. We obtained a p-value for each pathway (p_i). In order to determine the false positives we corrected the p-values using fdr (false discovery rate with BY algorithm -Benjamini and Yekutieli)

v) Cell subpopulation isolation, extraction and cell culture

The extraction and isolation of the PC3 subpopulations was carried out previously an published in Toni Celià-Terrassa T *et al.*

In brief, PC-3/M and PC-3/S cells were clonally derived from the human cell line PC-3 (Kaighn ME *et al.* 1979). Both sublines carry the integrated firefly luciferase gene coding region cloned in the Superluc pRC/CMV vector (Invitrogen).

The PC-3/M clone was selected by limiting dilution from PC-3/M, isolated from liver metastases produced in nude mice subsequent to intrasplenic injection of PC-3 cells (Kozlowski JM *et al.* 1984).

PC-3/S cells were selected by limiting dilution from parental PC-3 cells. Cells were grown at 37°C in a 5% CO2 atmosphere in complete RMPI 1640 supplemented with 200 µg/ml Geneticin (Sigma-Aldrich) to maintain the chromosomal integration of the luciferase gene. TSU-Pr1 and B2 cells were maintained at 37°C in a 5% CO2 atmosphere in complete DMEM.

All media were supplemented with 2 mM l-glutamine, 100 U/ml penicillin, 100 µg/ml streptomycin, and 10% FBS. Unless otherwise indicated, media and sera were from PAA.

PC-3/M and PC-3/S cells were cultivated separately using the media composition as is indicated in Aguilar et al 2016. Cells were cultured at 37°C in a 5% CO 2 atmosphere in RPMI 1640 media (Sigma-Aldrich or Biowest) supplemented with 10 mM glucose and 2 mM glutamine (unless otherwise indicated), 10% Fetal Bovine Serum (FBS) (PAA Laboratories), 1% pyruvate (1 mM) (Biological Industries), 1% streptomycin (100 µg/mL) / penicillin (100 units/mL) (Gibco) and 1% nonessential amino acids (Biological Industries).

Supplementary references:

- Aguilar E, Marin de Mas I, Zodda E, Marin S, Morrish F, Selivanov V, Meca-Cortés O, Delowar H, Pons M, Izquierdo I, Celià-Terrassa A, de Atauri P, Centelles J J, Hockenbery D, Thomson T M, Cascante M (2016) Metabolic landscape and vulnerabilities of metastatic prostate epithelial cancer stem cells independent of epithelial-mesenchymal transition. *Stem Cells.* **34(5)**:1163-76.
- Bardot V, Dutrillaux AM, Delattre JY, Vega F, Poisson M, Dutrillaux B, Luccioni C. (1994) Purine and pyrimidine metabolism in human gliomas: relation to chromosomal aberrations. *Br J Cancer.* **70(2)**:212-8.
- Brézillon S, Pietraszek K, Maquart FX, Wegrowski Y. (2013) Lumican effects in the control of tumour progression and their links with metalloproteinases and integrins. *FEBS J.* **280(10)**:2369-81.

- Celià-Terrassa T, Meca-Cortés O, Mateo F, de Paz AM, Rubio N, Arnal-Estapé A, Ell BJ, Bermudo R, Díaz A, Guerra-Rebollo M, Lozano JJ, Estarás C, Ulloa C, Álvarez-Simón D, Milà J, Vilella R, Paciucci R, Martínez-Balbás M, de Herreros AG, Gomis RR, Kang Y, Blanco J, Fernández PL, Thomson TM. Epithelial-mesenchymal transition can suppress major attributes of human epithelial tumor-initiating cells. J Clin Invest. 2012; **122(5):**1849-68. PMID: 22505459.
- Kaighn ME, Narayan KS, Ohnuki Y, Lechner JF, Jones LW. (1979) Establishment and characterization of a human prostatic carcinoma cell line (PC-3). Invest Urol. **17(1)**:16–23.
- Kozlowski JM, Fidler IJ, Campbell D, Xu ZL, Kaighn ME, Hart IR. (1984) Metastatic behavior of human tumor cell lines grown in the nude mouse. Cancer Res. **44(8):**3522–3529
- Munetsuna E, Kawanami R, Nishikawa M, Ikeda S, Nakabayashi S, Yasuda K, Ohta M, Kamakura M, Ikushiro S, Sakaki T. (2014) Anti-proliferative activity of 25-hydroxyvitamin D3 in human prostate cells. *Mol Cell Endocrinol.* **382(2)**:960-70.
- Peracaula R, Tabarés G, Royle L, Harvey DJ, Dwek RA, Rudd PM, de Llorens R. (2003) Altered glycosylation pattern allows the distinction between prostate-specific antigen (PSA) from normal and tumor origins. *Glycobiology* **13(6)**:457-70.
- Trump DL, Muindi J, Fakih M, Yu WD, Johnson CS. (2006) Vitamin D compounds: clinical development as cancer therapy and prevention agents. *Anticancer Res.* **26(4A)**:2551-6.
- Wang C, Chen MH, Schifano E, Wu J, Yan J. (2016) Statistical methods and computing for big data. *Stat Interface*. **9(4)**:399-414.
